# Association of systemic immune inflammatory index with all-cause and cause-specific mortality in hypertensive individuals: Results from NHANES

**DOI:** 10.3389/fimmu.2023.1087345

**Published:** 2023-02-02

**Authors:** Yang Cao, Pengxiao Li, Yan Zhang, Miaohan Qiu, Jing Li, Sicong Ma, Yudong Yan, Yi Li, Yaling Han

**Affiliations:** ^1^ The Department of Cardiology, General Hospital of Northern Theater Command, Shenyang, Liaoning, China; ^2^ The Department of Cardiology, Xijing Hospital, Air Force Medical University, Xi’an, Shanxi, China

**Keywords:** systemic immune-inflammation index, hypertension, population-based study, NHANES, cross-sectional study

## Abstract

**Background:**

The relationship between the systemic immune inflammatory index (SII) and the prognosis of hypertensive patients is unclear. This study aims to explore the association of SII with all-cause and cause-specific mortality in patients with hypertension.

**Methods:**

This study included 8524 adults with hypertension from the National Health and Nutritional Examination Surveys (NHANES) 2011–2018, and followed for survival through December 31, 2019. Cox proportional hazards models were used to investigate the associations between SII and mortality from all causes, cardiovascular disease (CVD), and cancer. Restricted cubic spline, piecewise linear regression, subgroup and sensitivity analyses were also used.

**Results:**

During a median follow-up of 4.58 years, 872 all-cause deaths occurred. After adjusting for covariates, higher SII was significantly associated with an elevated risk of CVD mortality. There was a 102% increased risk of CVD mortality per one-unit increment in natural log-transformed SII (lnSII) (P < 0.001). Consistent results were also observed when SII was examined as categorical variable (quartiles). The associations of SII with all-cause and cancer mortality were detected as U-shaped with threshold values of 5.97 and 6.18 for lnSII respectively. Below thresholds, higher SII was significantly associated with lower all-cause mortality (HR=0.79, 95%CI=0.64-0.97) and cancer mortality (HR=0.73, 95%CI=0.53-1.00). Above thresholds, SII was significantly positive associated with all-cause mortality (HR=1.93, 95%CI=1.55-2.40) and cancer mortality (HR=1.93, 95%CI=1.22-3.05). The results were robust in subgroup and sensitivity analyses.

**Conclusion:**

Higher SII (either as a continuous or categorical variable) were significantly associated with a higher risk of CVD mortality. The U-shaped associations were observed between SII and all-cause and cancer mortality.

## 1 Introduction

Hypertension is a major global public health challenge. It was estimated that 31.1% of the adult population worldwide (1.39 billion people) had hypertension in 2010, which approximately accounted for 10% of the world’s total healthcare expenditures (1). Meanwhile, hypertension is also an important interventionable risk factor for cardiovascular and cerebrovascular diseases (2, 3). It is of great meaning to identify modifiable factors for the delay or prevention of hypertension-related target organ damage.

Systemic immune-inflammation index (SII, platelets count × neutrophil/lymphocyte ratio), which was firstly proposed to evaluate the prognosis of hepatocellular carcinoma (4), has been recently reported as an excellent inflammatory marker associated with the prognosis of patients with various cardiovascular diseases (CVD) (5–8).

It is universally appreciated that low-grade inflammation plays an important role in initiating and maintaining elevated blood pressure (9, 10). Meanwhile, the cardiovascular risk of hypertensive patients persists even after blood pressure is effectively controlled. This further suggests the existence of residual cardiovascular risk, which could be partly attributed to underlying immune cell activation and chronic inflammation (11). Existing studies suggested that high SII is associated with increased carotid intima-media thickness and left ventricular hypertrophy in hypertensive patients (12–14). However, the relationship between SII and long-term clinical events, especially mortality, was not well studied in these patients.

To fill the knowledge gap, we aimed to explore the relationship between SII and all-cause and cause-specific mortality using a nationally-representative sample of adults (≥18 years) with hypertension in the United States (US).

## 2 Method

### 2.1 Study population

The National Health and Nutrition Examination Survey (NHANES) is a population-based cross-sectional survey designed to collect information on the health and nutrition of the U.S. household population (15). Data was collected through structured interviews at home, physical examination at a mobile center, and laboratory tests, and designed with multistage probability sampling. The protocol of NHANES was approved by the National Centre for Health Statistics (NCHS) ethics review board, and all participants had provided written informed consent.

**Figure 1:**
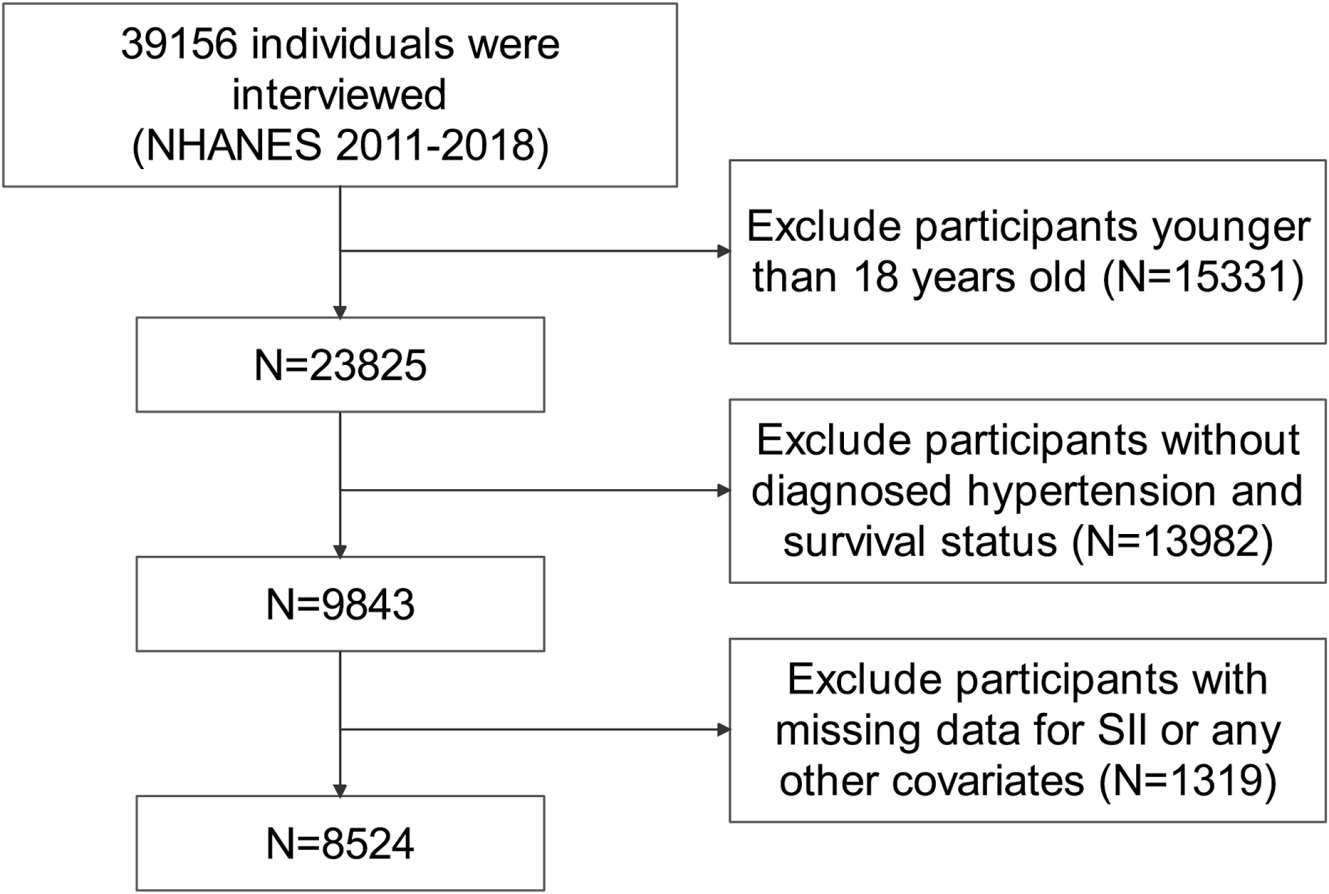
Flow diagram of the selection of eligible participants.

A total of 39156 individuals were identified from NHANES 2011−2018 data. We excluded subjects younger than 18 years of age (n=15331). In addition, subjects without diagnosed hypertension or survival status were further excluded (n=13982). Hypertension was determined by a combination of self-reported physician diagnosis, use of antihypertensive drugs and having a systolic blood pressure ≥140 or/and a diastolic blood pressure ≥90 mmHg. Moreover, we excluded individuals with missing values for SII or any other covariates (n=1319). Finally, 8524 subjects were enrolled in this study. The entire process of data selection is shown in **Figure 1**. All data used in this study is publicly available (https://www.cdc.gov/nchs/nhanes/) and weighted demographically for subsequent analysis.

### 2.2 Definition of systemic immune-inflammation index

The complete blood count is performed on the Coulter^®^ DxH 800 analyzer and supervised by trained medical personnel. The SII was calculated by platelet count ×neutrophil count/lymphocyte count and expressed as × 10^9^ cells/µl according to previous studies (4).

### 2.3 Outcome ascertainment

All-cause mortality was determined by the records of National Death Index (NDI) before 31 December 2019, which was linked with the NHANES datasets. Cause-specific mortality was ascertained by ICD-10. CVD death was defined as ICD-10 codes I00-I09, I11, I13, and I20-I51. Cancer death was defined as ICD-10 code C00-C97.

### 2.4 Assessment of covariates

We included various covariates that may affect the outcome. The age, sex, race, smoking status, medication use, and disease status were collected from standardized questionnaires in household interviews. Body mass index (BMI) was obtained through physical examinations in the mobile examination center. The laboratory indexes used included glycated hemoglobin (HbA1c), total cholesterol (TC), triglyceride (TG), creatinine (Cr), alanine aminotransferase (ALT), aspartate aminotransferase (AST), and estimated glomerular filtration rate (eGFR). The details of relevant definitions were shown in the **Supplementary Method**.

### 2.5 Statistical analyses

Considering that the NHANES adopted a complex, multistage, probability sampling design to select representative participants, we incorporate sample weights, clustering, and stratification in all analyses to obtain the whole national estimates. Continuous variables were presented as the weighted means ± standard error, and categorical variables were expressed as frequency and percentage. One-way ANOVA test, Kruskal-Wallis H test or Chi-squared test were employed to compare continuous or categorical variables in different SII quartile groups.

The associations between SII and the risk of all-cause mortality or cause-specific mortality were analyzed by multivariate Cox regression model to estimate hazard ratios (HRs) and 95% confidence interval (CI). Proportional hazard assumption was examined using Schoenfeld residual methods. Since the distribution of SII is specifically skewed to the right, SII values were natural log-transformed and grouped into four subgroups by its quartiles, which were respectively included in models in the form of continuous and categorical variables. We used the first quartile of lnSII (Q1) as the reference group, and assigned a median value to each category to assess the linear trend.

The baseline variables were included in the multivariable COX regression model if they showed p<0.1 in univariate analysis or were clinically related to clinical prognosis. A crude model without variables adjusted was developed first, and multivariates were adjusted in model 1, including sex, age, eth, BMI and smoke. We further adjusted related laboratory indicators including HbA1c, TC, Cr, AST and eGFR in model 2. For model 3, we additionally adjusted diabetes (DM), coronary heart disease (CHD) and chronic obstructive pulmonary disease (COPD).

In order to investigate dose-response associations between SII and mortality, restricted cubic spline (RCS) regression with the multivariable adjustment mentioned above was used. Likelihood ratio test was used to test nonlinearity. The number of nodes was determined based on the lowest value of the Akaike information criterion (AIC). If nonlinearity was detected, two-piecewise Cox proportional hazards regression models were constructed according to the inflection point.

Stratified analyses were also conducted by gender (male and female), race (White, Black, Mexican, Hispanic, and other races), smoke (never, former, and now), diabetes (no, yes, impaired fasting glycaemia, and impaired glucose tolerance), CHD (no and yes), COPD (no and yes). Potential interactions between multiple stratification factors and ln-SII were also examined. Considering that age is a strong prognostic factor in most cardiovascular diseases. The RCS stratified by age was also conducted.

To test the robustness of our findings, two sensitivity analyses were performed. First, participants who died within 2 years of follow-up were removed to exclude the potential reverse causality. Second, Multiple imputation was conducted for variates with missing values. Then, the relationship between SII and all-cause mortality was repeatedly verified in the 10 imputated complete datasets. Third, both diabetes and cancer were closely associated with low-grade inflammation and all-cause mortality. To eliminate their confounding effect, we exclude patients with diabetes and cancer to verify the association between SII and mortality again. In this study, a two-side p-value < 0.05 was considered statistically significant. All analyses were conducted using R (version 4.2.1).

## 3 Results

### 3.1 Baseline characteristics of study participants

A total of 8527 hypertensive individuals were finally included in our study, of which 50.4% were female, with an average age of 57.41 ± 0.27 years. The incidence of all-cause death was 10.2% among these participants during a median follow-up of 4.58 years. The weighted mean ± standard error of lnSII was 6.17 ± 0.01. The baseline characteristics according to the lnSII quartiles were shown in the **Table 1**. Participants with higher SII levels were more likely to be female, now smoker and White and combined with asthma and COPD; and had higher BMI, eGFR and lower AST (all P < 0.05). The baseline characteristics according to all-cause mortality were shown in the **Supplementary Table 1**.

**Table 1:** Baseline characteristics of patients with hypertension according to quartiles of lnSII in NHANES 2011–2018.

	Q1	Q2	Q3	Q4	P-value
Age, years	57.54 ± 0.48	56.76 ± 0.47	57.26 ± 0.48	58.05 ± 0.44	0.246
BMI, kg/m^2^	30.44 ± 0.17	30.81 ± 0.21	31.57 ± 0.22	31.81 ± 0.27	< 0.001
HbA1c, %	5.92 ± 0.03	5.95 ± 0.03	5.94 ± 0.03	5.99 ± 0.03	0.24
TC, mg/dl	194.44 ± 1.47	195.46 ± 1.55	193.55 ± 1.39	192.20 ± 1.33	0.228
TG, mg/dl	167.21 ± 4.55	173.09 ± 3.24	175.68 ± 4.05	166.72 ± 2.86	0.185
Cr, mg/dl	0.95 ± 0.01	0.94 ± 0.01	0.93 ± 0.01	0.97 ± 0.02	0.097
ALT, (IU/L)	27.75 ± 0.89	26.46 ± 0.59	26.26 ± 0.69	25.08 ± 0.67	0.074
AST, (IU/L)	28.48 ± 0.71	25.56 ± 0.36	25.62 ± 0.44	25.56 ± 0.61	0.004
eGFR, (ml/min/1.73 m^2^)	85.48 ± 0.70	85.11 ± 0.62	85.00 ± 0.77	83.20 ± 0.61	0.033
Sex, n (%)					0.005
Male	1130 (54.47)	1063 (51.31)	1033 (49.22)	1006 (46.05)	
Female	1001 (45.53)	1068 (48.69)	1098 (50.78)	1125 (53.95)	
Race, n (%)					< 0.001
White	563 (56.32)	751 (65.33)	912 (70.66)	1068 (75.66)	
Black	895 (23.67)	556 (12.87)	468 (10.15)	385 (7.99)	
Mexican	204 (5.76)	247 (6.31)	261 (6.58)	264 (6.33)	
Hispanic	189 (5.23)	232 (5.89)	234 (5.20)	188 (4.26)	
Other Races	280 (9.01)	345 (9.60)	256 (7.41)	226 (5.76)	
Smoke, n (%)					0.024
Never	1122 (51.95)	1143 (52.06)	1132 (51.64)	986 (46.52)	
Former	628 (32.23)	621 (30.47)	611 (29.51)	697 (32.76)	
Now	381 (15.82)	367 (17.47)	388 (18.85)	448 (20.72)	
Diabetes, n (%)					0.094
No	1251 (64.04)	1244 (64.13)	1246 (61.43)	1156 (58.62)	
IFG	120 (8.04)	131 (6.70)	146 (8.58)	139 (7.65)	
IGT	89 (3.72)	64 (3.08)	81 (3.09)	74 (3.48)	
DM	671 (24.19)	692 (26.10)	658 (26.90)	762 (30.25)	
Hyperlipidemia, n (%)					0.346
No	456 (19.96)	407 (18.81)	385 (17.28)	377 (17.32)	
Yes	1675 (80.04)	1724 (81.19)	1746 (82.72)	1754 (82.68)	
Coronary Heart Disease, n (%)					0.346
No	1966 (91.81)	1973 (93.91)	1973 (93.00)	1963 (92.83)	
Yes	165 (8.19)	158 (6.09)	158 (7.00)	168 (7.17)	
Asthma, n (%)					0.001
No	1841 (86.08)	1800 (85.83)	1764 (82.20)	1734 (80.84)	
Yes	290 (13.92)	331 (14.17)	367 (17.80)	397 (19.16)	
COPD, n (%)					0.011
No	2033 (95.30)	2029 (94.61)	2001 (93.25)	1931 (92.11)	
Yes	98 (4.70)	102 (5.39)	130 (6.75)	200 (7.89)	
Status, n (%)					< 0.001
Alive	1940 (92.26)	1972 (93.62)	1931 (92.90)	1809 (87.52)	
Death	191 (7.74)	159 (6.38)	200 (7.10)	322 (12.48)	

Data are expressed as mean ± stand error or frequency (percentage).

### 3.2 Associations between SII and mortality

As shown in **Table 2**, lnSII was significantly associated with an increased risk of all-cause mortality (HR=1.58, 95%CI=1.28-1.95) in the crude model. The result of univariate analysis of COX regression model was shown in **Supplementary Table 2**. After multivariable adjustment, the results remained robust and statistically significant, with model 1 (HR=1.41, 95%CI=1.20-1.67), model 2 (HR=1.38, 95%CI=1.17 = 1.62) and model 3 (HR= 1.34, 95%CI=1.14-1.59). Compared to the first quartile of SII, multivariate-adjusted HRs for patients in the fourth quartile tend to be higher, with model 1 (HR=1.41, 95%CI=1.10-1.81, P for trend<0.001), model 2 (HR=1.38, 95%CI=1.08-1.78, P for trend =0.001), model 3 (HR=1.33, 95%CI=1.03-1.71, P for trend =0.003). This statistically significant association was consistent for CVD mortality, but no similar result was seen for cancer mortality.

**Table 2:** Weighted association between SII and mortality.

	Q1	Q2	Q3	Q4	P for Trend	lnSII
All-cause Mortality						
Unadjusted	1.00	0.79 (0.63,0.98)	0.88 (0.66,1.16)	1.59 (1.23,2.07)	<0.001	1.58 (1.28,1.95)
Model1	1.00	0.80 (0.64,1.00)	0.86 (0.66,1.13)	1.41 (1.10,1.81)	<0.001	1.41 (1.20,1.67)
Model2	1.00	0.79 (0.64,0.99)	0.87 (0.66,1.16)	1.38 (1.08,1.78)	0.001	1.38 (1.17,1.62)
Model3	1.00	0.76 (0.61,0.95)	0.85 (0.65,1.13)	1.33 (1.03,1.71)	0.003	1.34 (1.14,1.59)
CVD Mortality						
Unadjusted	1.00	0.95 (0.57,1.57)	1.17 (0.66,2.08)	2.45 (1.51,3.96)	<0.001	2.44 (1.78,3.33)
Model1	1.00	0.99 (0.58,1.68)	1.18 (0.64,2.19)	2.28 (1.34,3.87)	<0.001	2.14 (1.60,2.88)
Model2	1.00	0.98 (0.57,1.67)	1.15 (0.61,2.17)	2.22 (1.30,3.77)	<0.001	2.01 (1.51,2.66)
Model3	1.00	0.98 (0.56,1.71)	1.18 (0.62,2.26)	2.25 (1.28,3.97)	<0.001	2.02 (1.51,2.69)
Cancer Mortality						
Unadjusted	1.00	0.55 (0.32,0.94)	0.42 (0.25,0.73)	0.83 (0.54,1.29)	0.471	0.89 (0.61,1.30)
Model1	1.00	0.54 (0.31,0.92)	0.40 (0.24,0.67)	0.68 (0.43,1.08)	0.149	0.82 (0.61,1.12)
Model2	1.00	0.55 (0.32,0.95)	0.42 (0.25,0.69)	0.69 (0.44,1.09)	0.155	0.84 (0.62,1.13)
Model3	1.00	0.52 (0.31,0.90)	0.39 (0.24,0.65)	0.64 (0.41,1.01)	0.092	0.81 (0.61,1.07)

Data are presented as HR (95%CI).

Model 1: adjust for sex, age, eth, BMI, smoke.

Model 2: adjust for sex, age, eth, BMI, smoke, HbA1c, TC, Cr, AST, eGFR.

Model 3: adjust for sex, age, eth, BMI, smoke, HbA1c, TC, Cr, AST, eGFR, DM, CHD, COPD.

### 3.3 Dose-response relationship between SII and mortality

**Figure 2:**
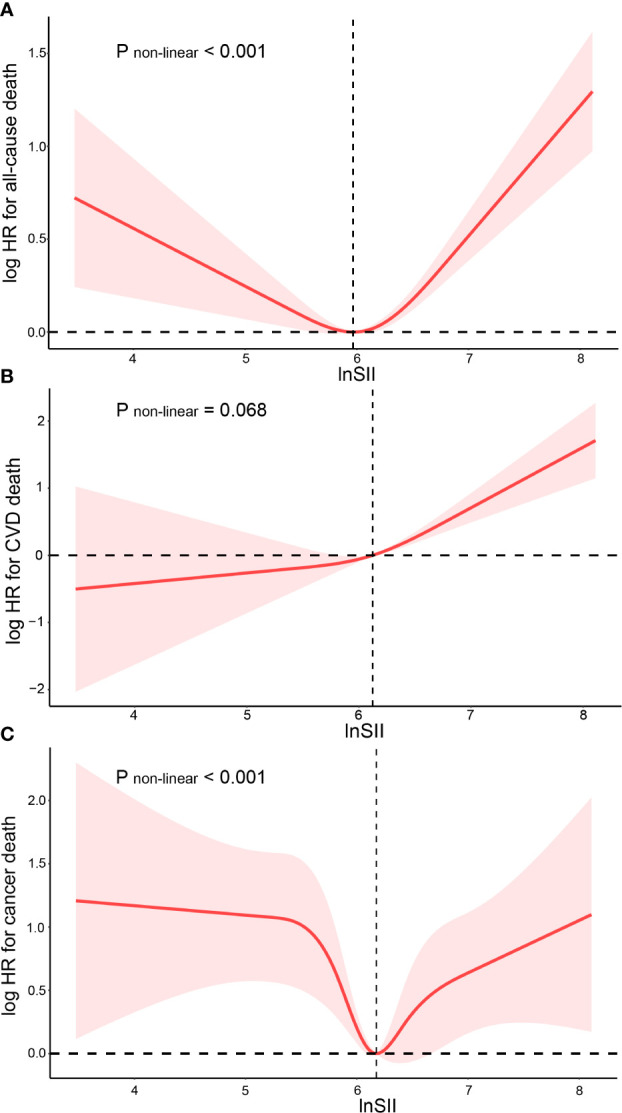
Restricted cubic spline fitting for the association between SII with mortality. Association of SII levels with the all-cause **(A)**, CVD mortality **(B)** and cancer mortality **(C)**.

As demonstrated in **Figure 2**, after adjusting for multiple potential confounders, the nonlinear associations between SII and all-cause mortality or cancer mortality were statistically significant (P<0.05), indicating the necessity to employ RCS to fit the Cox regression model for further evaluation. However, there was no nonlinear association between SII and CVD mortality (P=0.068). The two-piecewise linear regression in **Table 3** revealed that the risk of all-cause mortality first decreased and reached the minimum at the ln-SII value of 5.97 (SII = 391.5×10^9^ cells/µl) and then increased as SII increased (HR=0.79, 95%CI=0.64-0.97; HR=1.93, 95%CI=1.55,2.40). Similarly, risk of CVD mortality fell with rising lnSII up to 6.18 (SII = 481.1×10^9^ cells/µl), then ascended with greater level of SII (HR=0.73, 95%CI=0.53-1.00; HR=1.93, 95%CI=1.22-3.05).

**Table 3:** Nonlinearity addressed through two-piecewise linear model.

	All-cause mortality	P-value	Cancer mortality	P-value
Threshold value	5.97		6.18	
< Threshold value	0.79 (0.64,0.97)	0.023	0.73 (0.53, 1.00)	0.053
> Threshold value	1.93 (1.55,2.40)	<0.001	1.93 (1.22,3.05)	0.004

Hazard ratios were calculated based on multivariable Cox proportional hazards regression model adjusted for sex, age, eth, BMI, smoke, HbA1c, TC, Cr, AST, eGFR, DM, CHD, COPD. HR, hazard ratio; CI, confidence interval.

### 3.4 Subgroup analyses and sensitivity analyses

**Figure 3:**
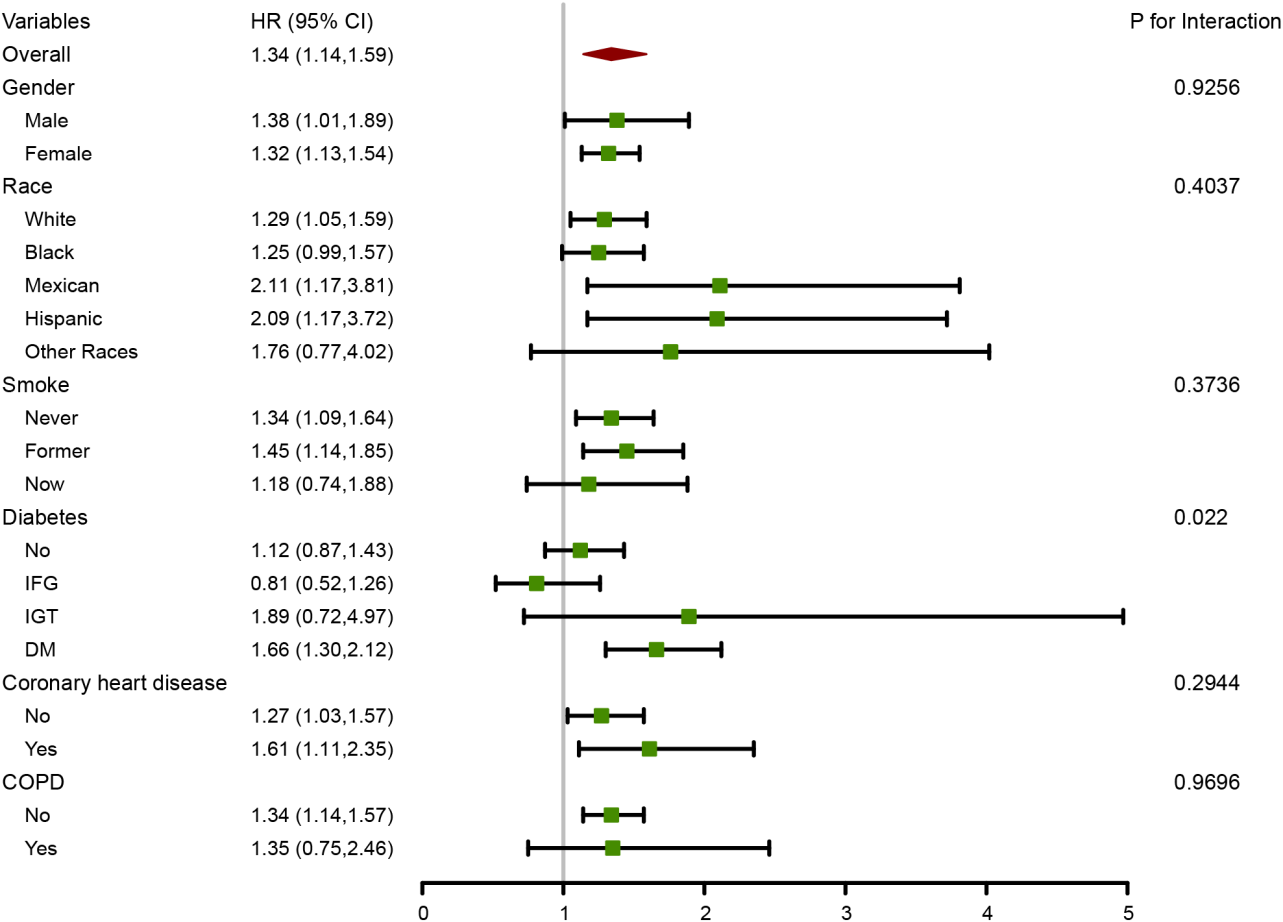
Forest plot for subgroup analysis of association between lnSII and all-cause mortality. Hazard ratios (HRs) were calculated using multivariate Cox regression models adjusted for the variables listed in the fully adjusted model except for the variable used for stratification.

As shown in **Figure 3**, Subgroup analyses were conducted to determine whether demographic characteristics and comorbidities could account for the association between ln-SII and all-cause mortality. Consistent results were observed when analyses were stratified by sex, race, smoking status, coronary heart disease and COPD (all P for interaction > 0.05). A significant interaction was found between SII and diabetes (P for interaction < 0.05). The associations between lnSII and all-cause and cause-specific mortality were examined in different age subgroups. **Supplementary Table 3** demonstrated that the associations were generally consistent with those of the whole population. It deserved to be noted that there was a 207% increased risk of CVD mortality per one-unit increment in lnSII in hypertensive patients younger than 60 years old. The RCS results stratified by age in the **Supplementary Figure 1** demonstrated that compared with the whole population, there was no U-shaped relationship between lnSII and the risk of cancer mortality in hypertensive patients younger than 60 years old. In hypertensive patients older than 60 years old, the association between lnSII and CVD mortality showed a U-shaped relationship, rather than a linear relationship in the whole population.

In the sensitivity analyses, the results did not significantly change after excluding participants who died within the first 2 years of follow-up (**Supplementary Table 4**). The proportion of missing data and diagnosis of multiple imputation were provided in the **Supplementary Table 5** and **Supplementary Figure 2**. **Supplementary Figure 3** demonstrated that the association between SII and all-cause mortality was consistent in the 10 imputated datasets, which eliminated the impact of missing data on the results to a certain extent. As shown in **Supplementary Table 6**, SII was still associated with an increased risk of all-cause mortality after excluding participants with diabetes and cancer. It is worth mentioning that the correlation between SII and CVD mortality was significantly higher after excluding patients with diabetes and cancer, suggesting that SII may be more closely related to cardiovascular mortality.

## 4 Discussion

In this nationally representative cross-sectional study, we found U-shaped relationships between SII levels and the risk of all-cause mortality and cancer mortality, with either too low or too high concentrations associated with increased risk. The levels of lnSII (SII) with the lowest risk of all-cause mortality and cancer-mortality were 5.97 (391.5×10^9^ cells/µl) and 6.18 (481.1×10^9^ cells/µl) respectively. These findings were consistent after various sensitivity and stratified analyses. In addition, the relationship between SII and CVD mortality tends to be linearly correlated, with higher SII associated with greater risk of CVD mortality. As far as we know, this is the first study to examine the association between the SII and all-cause and cause-specific mortality among hypertensive populations.

A growing body of evidence suggests that the chronic over-activation of immune cells and subsequent low-grade inflammation might be the likely culprits of hypertension and its deadly complications (16). In recent years, genome-wide association studies (GWAS) have provided powerful insights into the genetic basis of hypertension (17–20). A pooled study of multiple GWAS showed that a considerable proportion of SNPS associated with hypertension were also directly or indirectly involved in inflammation and immunity (21). Multiple clinical and epidemiologic studies have demonstrated that many plasma inflammatory markers such as C-reactive protein, high-sensitivity C-reactive protein, interleukin-6 and interleukin-1β are often elevated in patients with hypertension and associated with prognosis (22–25). While there are no large clinical trials focusing on anti-inflammatory treatment of hypertension to the best of our knowledge, a secondary analysis of the CANTOS trial has demonstrated the patients in the highest quartile of blood pressure tend to have greater benefit from anti-inflammatory therapy with canakinumab (26). This hints at a possible cumulative effect between the level of inflammation and blood pressure.

SII is considered to be able to comprehensively evaluate the level of immunity and inflammation status. In a meta-analysis involving 152,996 patients, higher SII was associated with an increased risk of nearly all CVD subtypes, including myocardial infarction, ischemic or hemorrhagic stroke, and peripheral arterial disease (27). Basic research has also confirmed the involvement of SII components in hypertension. First, as regulators of cardiovascular inflammation and first responders of the immune system, neutrophils were elevated in hypertensive patients (28, 29). In hypertensive animal models, depletion of neutrophils could partly attenuate hypertension (30, 31). Meanwhile, neutrophils are also involved in hypertensive end-organ damage including heart, vasculature, and kidney (31–33). In recent years, Neutrophil extracellular traps (NETs) have been considered as the forefront of research in neutrophil biology, which established a novel link between inflammation, innate immunity, oxidative stress, and cardiovascular disease (34, 35). Plasma levels of NETs were elevated in patients with essential hypertension and associated with the accelerated formation of atherosclerosis and thrombosis (36, 37). Notably, there is compelling evidence that NETs and comorbid hypertension correlate with the severity and outcomes in patients with COVID-19 (38, 39). Second, a bi-directional mendelian randomization study revealed that platelet counts have a causal effect on hypertension in the general population (40). The relationship between abnormal platelet activation and the risk of thrombosis in hypertensive patients is also well-established (41, 42). Third, there has been a plethora of studies focusing on the mechanisms of lymphocyte involvement in hypertension and demonstrating that T cells are of particular importance in the pathogenesis (11, 43). In recent years, the gut microbiome has also been shown to be involved in the development and progression of hypertension through T-lymphocyte-mediated inflammatory responses (44).

As a composite index, SII complicates the analysis of the U-shaped relationship in this study. Different lymphocyte subsets play different roles in hypertension, among which B1 cells and Treg cells play a potential protective role (11). While participating in the inflammatory response, low platelets and lymphocytes also mean an increased risk of bleeding and reduced immunity. Those functional differences and diversity may be at the root of the U-shaped relationship. Meanwhile, platelets could promote the genesis and metastasis of tumor through various crosstalk with cancer cells (45, 46). Lymphocytes exert protective effects by inducing cytotoxic cell death and inhibiting tumor cell proliferation and migration (47). The specific functions of platelets and lymphocytes in the oncology field might explain the absence of a U-shaped relationship between SII and CVD death.

In previous reports, SII often showed a linear relationship with the prognosis of various diseases (6, 8, 12–14). Yet U-shaped relationships tend to better reflect the true role of immune inflammation levels in disease progression and were reported in studies of hepatic steatosis, gastric cancer and congestive heart failure (7, 48, 49). The inflection point in the U-shaped relationship represents the SII value with the lowest risk of death, which can help us to accurately identify the residual cardiovascular risk in hypertensive patients and further develop personalized treatment plan in clinical practice.

There are several strengths and limitations in the current study. We conducted analyses using a large, nationally representative sample and adjusted for demographic, examinational, and laboratory covariates to ensure that the associations are credible and generalizable. Multiple sensitivity and subgroup analyses further confirmed the robust of our results. Certain limitations should be considered as well. First, the observational study design could not establish authentic causality, although we exclude participants who died within 2 years of follow-up in the subgroup analysis. Second, SII was measured only once in our study, which may underestimate the association. Third, there is no detailed information on stages of hypertension in our study, so it is not able to explore whether the association between SII and prognosis differs in patients with different severity of hypertension.

## 5 Conclusion

In conclusion, we found that U shaped relationships between SII and all-cause mortality and cancer-mortality with threshold values of 5.97 and 6.18 for ln-SII respectively. Higher concentrations of SII was linearly associated with elevated risk of CVD mortality. These results support the independent prognostic value of SII for hypertensive patients.

## Data availability statement

The datasets presented in this study can be found in online repositories. The names of the repository/repositories and accession number(s) can be found below: The datasets analyzed in this study are publicly available and can be found here: https://www.cdc.gov/nchs/nhanes/.

## Author contributions

YH and YL directed the study and revised the manuscript for important intellectual content. YC and YZ conceived and designed the methodology for the study. YY, MQ, SM and JL collected samples. YC and PL conducted statistical analysis and models development. YC drafted the manuscripts. All authors contributed to the article and approved the submitted version.
