# Supplementary material for: Association of systemic immune inflammatory index with all-cause and cause-specific mortality in hypertensive individuals: Results from NHANES
Source: Front Immunol. 2023 Feb 2;14:1087345. doi: 10.3389/fimmu.2023.1087345 (PMC9932782; doi:10.3389/fimmu.2023.1087345)
Supplement: Supplementary file 1 [file DataSheet_1.docx]

Supplementary Material

# Supplementary methods

Smoking status were classified as never smoking (<100 cigarettes in lifetime), former smoking (>100 cigarettes in life and smoke not at all now), and now smoking (>100 cigarettes in lifetime).

Diabetes was diagnosed by:

- Self-reported doctor diagnosis of diabetes.
- Glycohemoglobin HbA1c > 6.5%.
- Fasting glucose ≥7.0 mmol/l.
- Random blood glucose ≥11.1 mmol/l.
- Two-hour OGTT blood glucose ≥ 11.1 mmol/l.
- Use of diabetes medication or insulin.

Impaired Fasting Glycaemia (IFG) was diagnosed by:

- 6.1 mmol/l ≤ Fasting glucose ≤ 7.0 mmol/l.

Impaired Glucose Tolerance (IGT) was diagnosed by:

- 7.8 mmol/l ≤ Two-hour OGTT blood glucose ≤ 11.1 mmol/l.

Hyperlipidemia was diagnosed by:

- Hypertriglyceridemia: TG ≥ 150mg/dl.
- Hypercholesterolemia: TC ≥ 200mg/dl, LDL ≥ 130mg/dl.
- Low HDL: HDL < 40mg/dl (male),50mg/dl (female).
- Use of lipid-lowering drugs.

Coronary heart disease was diagnosed by:

- Self-reported doctor diagnosis of coronary heart disease.

Asthma was diagnosed by:

- Self-reported doctor diagnosis of asthma.
- Use of antiasthmatic drug.
- Subjects who are younger than 40 years, have no smoke, chronic bronchitis and emphysema, and use the medications including selective phosphodiesterase-4 inhibitors, mast cell stabilizers, leukotriene modifiers and inhaled corticosteroids.

COPD was diagnosed by:

- FEV1/FVC < 0.7 Post-Bronchodilator.
- Self-reported doctor diagnosis of emphysema.
- Subjects who are older than 40 years, have a history of chronic bronchitis, and use the medications including selective phosphodiesterase-4 inhibitors, mast cell stabilizers, leukotriene modifiers and inhaled corticosteroids.

# Supplementary Tables

## Supplementary Table 1. Baseline characteristics according to all-cause mortality

| Variables | Alive (7652) | Death (872) | P-value |
| --- | --- | --- | --- |
| Age, years | 56.25 ± 0.26 | 69.88 ± 0.59 | < 0.001 |
| BMI, kg/m2 | 31.33 ± 0.14 | 29.87 ± 0.30 | < 0.001 |
| HbA1c, % | 5.93 ± 0.02 | 6.17 ± 0.06 | < 0.001 |
| TC, mg/dl | 194.71 ± 0.96 | 184.44 ± 1.64 | < 0.001 |
| TG, mg/dl | 171.20 ± 2.28 | 166.36 ± 5.60 | 0.424 |
| Cr, mg/dl | 0.93 ± 0.01 | 1.17 ± 0.04 | < 0.001 |
| ALT, (IU/L) | 26.46 ± 0.34 | 24.53 ± 1.69 | 0.255 |
| AST, (IU/L) | 25.85 ± 0.24 | 29.83 ± 1.74 | 0.026 |
| eGFR, (ml/min/1.73 m2) | 86.15 ± 0.38 | 68.33 ± 1.05 | < 0.001 |
| lnSII | 6.16 ± 0.01 | 6.30 ± 0.03 | < 0.001 |
| Sex, n (%) |  |  | 0.958 |
| Male | 3749 (49.95) | 483 (50.06) |  |
| Female | 3903 (50.05) | 389 (49.94) |  |
| Race, n (%) |  |  | < 0.001 |
| White | 2811 (66.81) | 483 (77.50) |  |
| Black | 2094 (13.22) | 210 (11.29) |  |
| Mexican | 906 (6.50) | 70 (3.83) |  |
| Hispanic | 789 (5.34) | 54 (2.71) |  |
| Other Races | 1052 (8.13) | 55 (4.67) |  |
| Smoke, n (%) |  |  | < 0.001 |
| Never | 4038 (51.54) | 345 (38.06) |  |
| Former | 2204 (30.36) | 353 (40.35) |  |
| Now | 1410 (18.09) | 174 (21.59) |  |
| Diabetes, n (%) |  |  | < 0.001 |
| No | 4505 (63.36) | 392 (45.92) |  |
| IFG | 492 (7.82) | 44 (6.92) |  |
| IGT | 263 (3.14) | 45 (5.34) |  |
| DM | 2392 (25.68) | 391 (41.82) |  |
| Hyperlipidemia, n (%) |  |  | 0.158 |
| No | 1480 (18.46) | 145 (15.83) |  |
| Yes | 6172 (81.54) | 727 (84.17) |  |
| Coronary Heart Disease, n (%) |  |  | < 0.001 |
| No | 7161 (93.86) | 714 (82.97) |  |
| Yes | 491 ( 6.14) | 158 (17.03) |  |
| Asthma, n (%) |  |  | 0.715 |
| No | 6402 (83.48) | 737 (84.16) |  |
| Yes | 1250 (16.52) | 135 (15.84) |  |
| COPD, n (%) |  |  | < 0.001 |
| No | 7244 (94.62) | 750 (83.90) |  |
| Yes | 408 ( 5.38) | 122 (16.10) |  |

Abbrevation: body mass index, BMI; glycated hemoglobin, HbA1c; total cholesterol, TC; triglyceride, TG; creatinine, Cr; alanine aminotransferase, ALT; aspartate aminotransferase, AST; estimated glomerular filtration rate, eGFR; impaired fasting glycaemia, IFG; impaired glucose tolerance, IGT; diabetes, DM; chronic obstructive pulmonary disease, COPD.

## Supplementary Table 2. Univariate analysis of COX regression model

| Variables | HR (95%CI) | P-value |
| --- | --- | --- |
| Age | 1.09 (1.07,1.10) | <0.001 |
| BMI | 0.98 (0.96,0.99) | <0.001 |
| HB | 0.77 (0.73,0.81) | <0.001 |
| HbA1c | 1.16 (1.10,1.23) | <0.001 |
| TC | 0.99 (0.99,1.00) | <0.001 |
| TG | 1.00 (1.00,1.00) | 0.275 |
| Cr | 1.32 (1.22,1.43) | <0.001 |
| ALT | 0.99 (0.98,1.01) | 0.367 |
| AST | 1.00 (1.00,1.01) | <0.001 |
| eGFR | 0.97 (0.97,0.97) | <0.001 |
| lnSII | 1.58 (1.28,1.95) | <0.001 |
| Sex |  |  |
| Male | 1 |  |
| Female | 1.00 (0.85,1.17) | 1.000 |
| Race |  |  |
| White | 1 |  |
| Black | 0.76 (0.60,0.95) | 0.018 |
| Mexican | 0.53 (0.37,0.75) | <0.001 |
| Hispanic | 0.47 (0.34,0.65) | <0.001 |
| Other Races | 0.56 (0.41,0.78) | <0.001 |
| Smoke |  |  |
| Never | 1 |  |
| Former | 1.72 (1.39,2.15) | <0.001 |
| Now | 1.52 (1.13,2.04) | 0.006 |
| Diabetes |  |  |
| No | 1 |  |
| IFG | 1.39 (0.91,2.11) | 0.125 |
| IGT | 1.68 (1.06,2.67) | 0.028 |
| DM | 2.28 (1.81,2.88) | <0.001 |
| Hyperlipidemia |  |  |
| No | 1 |  |
| Yes | 1.17 (0.91,1.51) | 0.220 |
| Coronary Heart Disease |  |  |
| No | 1 |  |
| Yes | 3.21 (2.48,4.14) | <0.001 |
| Asthma |  |  |
| No | 1 |  |
| Yes | 0.96 (0.74,1.26) | 0.792 |
| COPD |  |  |
| No | 1 |  |
| Yes | 2.71 (2.11,3.47) | <0.001 |

## Supplemantary Table 3. Weighted association between lnSII and mortality stratified by age

|  | Age <60 | Age ≥60 | P for Interaction |
| --- | --- | --- | --- |
| All-cause Mortality | 1.56 (1.00,2.43) | 1.32 (1.11,1.56) | 0.368 |
| CVD Mortality | 3.07 (1.87, 5.04) | 1.82 (1.33,2.50) | 0.150 |
| Cancer Mortality | 1.00 (0.51, 1.95) | 0.85 (0.60,1.20) | 0.968 |

Data are expressed as HR (95%CI)

## Supplemantary Table 4. Hazard ratios (95% CI) of all-cause and specific cause mortality according to quartiles of lnSII among patients with hypertension after excluding participants who died within two years of follow-up (n=8256)

|  | Q1  <5.77 | Q2  5.77-6.12 | Q3  6.13-6.47 | Q4  >6.48 | P for Trend | lnSII |
| --- | --- | --- | --- | --- | --- | --- |
| All-cause Mortality |  |  |  |  |  |  |
| Unadjusted | 1.00 | 0.75 (0.57,0.98) | 0.78 (0.59,1.03) | 1.45 (1.08,1.95) | 0.004 | 1.47 (1.14,1.89) |
| Model1 | 1.00 | 0.76 (0.59,0.98) | 0.78 (0.60,1.01) | 1.28 (0.96,1.71) | 0.025 | 1.33 (1.09,1.63) |
| Model2 | 1.00 | 0.76 (0.59,0.98) | 0.80 (0.61,1.05) | 1.29 (0.97,1.72) | 0.025 | 1.32 (1.09,1.60) |
| Model3 | 1.00 | 0.74 (0.57,0.96) | 0.79 (0.60,1.04) | 1.26 (0.93,1.70) | 0.036 | 1.31 (1.07,1.60) |
| CVD Mortality |  |  |  |  |  |  |
| Unadjusted | 1.00 | 0.89 (0.51,1.58) | 0.96 (0.53,1.75) | 2.33 (1.37,3.98) | <0.001 | 2.24 (1.52,3.32) |
| Model1 | 1.00 | 0.93 (0.51,1.68) | 0.98 (0.51,1.87) | 2.15 (1.19,3.88) | 0.002 | 2.00 (1.39,2.88) |
| Model2 | 1.00 | 0.92 (0.50,1.70) | 0.98 (0.50,1.90) | 2.12 (1.18,3.79) | 0.001 | 1.87 (1.35,2.60) |
| Model3 | 1.00 | 0.92 (0.49,1.73) | 1.01 (0.50,2.01) | 2.15 (1.15,4.03) | 0.002 | 1.90 (1.36,2.64) |
| Cancer Mortality |  |  |  |  |  |  |
| Unadjusted | 1.00 | 0.41 (0.21,0.81) | 0.40 (0.21,0.75) | 0.82 (0.46,1.46) | 0.689 | 0.88 (0.53,1.48) |
| Model1 | 1.00 | 0.39 (0.20,0.77) | 0.38 (0.21,0.68) | 0.67 (0.38,1.20) | 0.379 | 0.82 (0.53,1.25) |
| Model2 | 1.00 | 0.40 (0.20,0.80) | 0.39 (0.22,0.69) | 0.69 (0.38,1.23) | 0.397 | 0.84 (0.55,1.29) |
| Model3 | 1.00 | 0.38 (0.19,0.75) | 0.37 (0.21,0.65) | 0.64 (0.35,1.16) | 0.318 | 0.82 (0.55,1.22) |

Data are presented as HR (95%CI)

Model 1: adjust for sex, age, eth, BMI, smoke

Model 2: adjust for sex, age, eth, BMI, smoke, HbA1c, TC, Cr, AST, eGFR

Model 3: adjust for sex, age, eth, BMI, smoke, HbA1c, TC, Cr, AST, eGFR, DM, CHD, COPD

## Supplementary Table 5. Missing of variables

| Variables | Missing | Missing proportion |
| --- | --- | --- |
| HbA1c | 773 | 7.85% |
| ALT | 969 | 9.84% |
| AST | 975 | 9.91% |
| Cr | 964 | 9.79% |
| TG | 970 | 9.85% |
| TC | 970 | 9.85% |
| BMI | 535 | 5.44% |
| SII | 776 | 7.88% |
| eGFR | 964 | 9.79% |
| Hyperlipidemia | 336 | 3.41% |
| DM | 24 | 0.24% |
| Coronary Heart Disease | 122 | 1.24% |
| Smoke | 28 | 0.28% |
| COPD | 64 | 0.65% |

## Supplementary Table 6. Hazard ratios (95% CI) of all-cause and specific cause mortality according to quartiles of lnSII among patients with hypertension after excluding participants with type 2 diabetes and cancer (n=4958)

|  | Q1  <5.77 | Q2  5.77-6.12 | Q3  6.13-6.47 | Q4  >6.48 | P for Trend | lnSII |
| --- | --- | --- | --- | --- | --- | --- |
| All-cause Mortality |  |  |  |  |  |  |
| Unadjusted | 1.00 | 0.74 (0.46,1.19) | 1.02 (0.69,1.51) | 1.61 (1.08,2.40) | 0.002 | 1.45 (1.02,2.06) |
| Model1 | 1.00 | 0.75 (0.46,1.23) | 1.05 (0.72,1.53) | 1.52 (1.02,2.26) | 0.005 | 1.34 (0.99,1.81) |
| Model2 | 1.00 | 0.79 (0.49,1.29) | 1.09 (0.76,1.55) | 1.51 (1.03,2.22) | 0.005 | 1.32 (1.00,1.73) |
| Model3 | 1.00 | 0.78 (0.48,1.28) | 1.07 (0.75,1.55) | 1.48 (1.00,2.18) | 0.007 | 1.30 (0.99,1.72) |
| CVD Mortality |  |  |  |  |  |  |
| Unadjusted | 1.00 | 0.74 (0.32,1.74) | 1.58 (0.65,3.85) | 3.39 (1.56,7.37) | <0.001 | 2.70 (1.68,4.32) |
| Model1 | 1.00 | 0.83 (0.34,2.01) | 1.83 (0.78,4.28) | 3.70 (1.69,8.08) | <0.001 | 2.55 (1.70,3.84) |
| Model2 | 1.00 | 0.84 (0.35,2.01) | 1.87 (0.84,4.17) | 3.83 (1.79,8.17) | <0.001 | 2.55 (1.71,3.82) |
| Model3 | 1.00 | 0.82 (0.33,2.05) | 1.85 (0.81,4.25) | 3.70 (1.74,7.86) | <0.001 | 2.55 (1.71,3.79) |

We firstly exclude participants with type 2 diabetes (n = 2783) from the whole population (n = 8524). Then the patients indicating in the questionnaire that they had not been diagnosed with cancer or malignancy (n = 4958) were included for the analysis. Data are presented as HR (95%CI).

Model 1: adjust for sex, age, eth, BMI, smoke

Model 2: adjust for sex, age, eth, BMI, smoke, HbA1c, TC, Cr, AST, eGFR

Model 3: adjust for sex, age, eth, BMI, smoke, HbA1c, TC, Cr, AST, eGFR, CHD, COPD

# Supplementary Figures

## Supplementary Figure 1. Restricted cubic spline fitting for the association between SII with mortality stratified by age


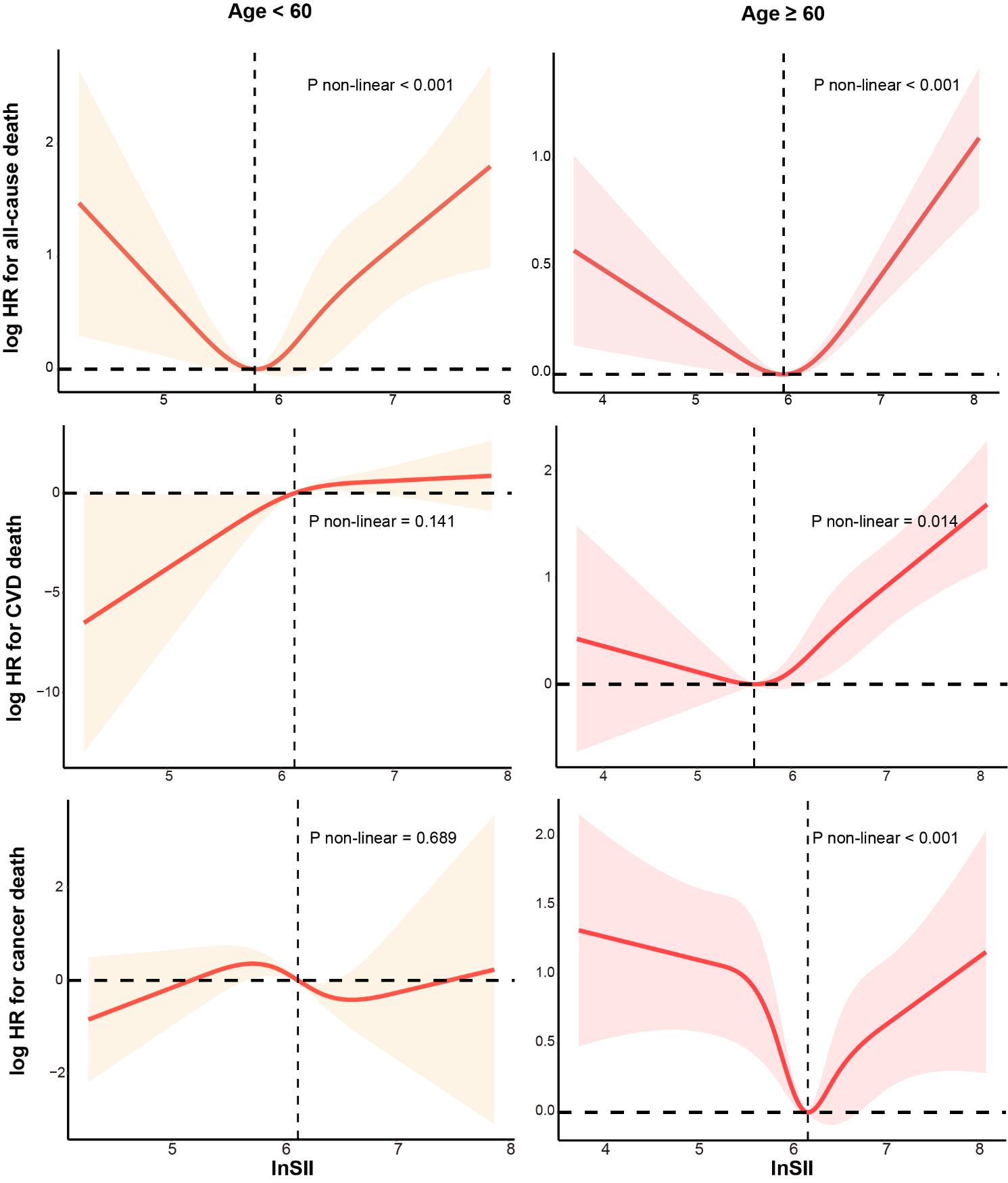


## Supplementary Figure 2. The distribution of data before and after imputation


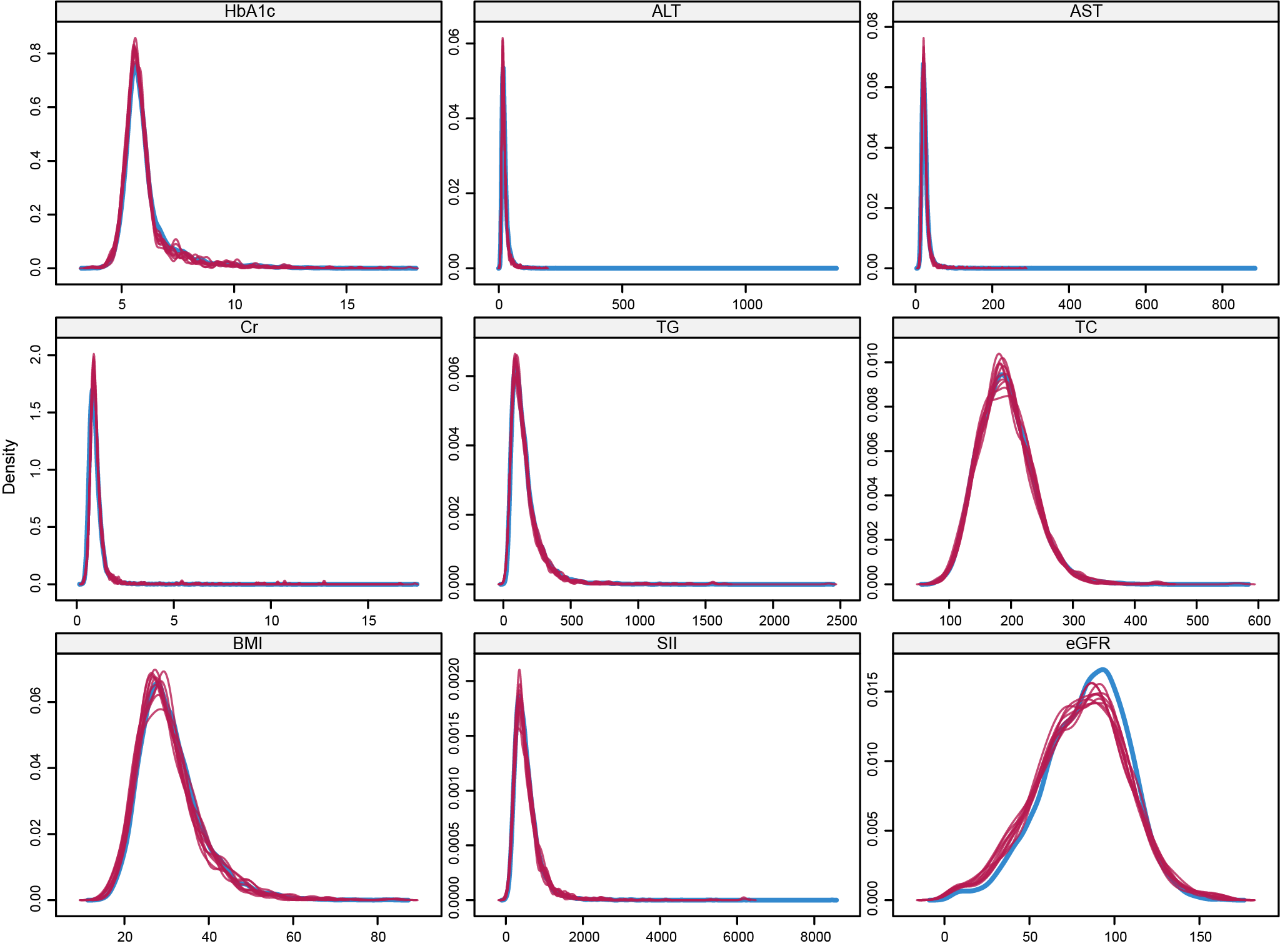


The blue line represents the original distribution of the non imputated data, and the red lines represent the distribution of the imputated data.

## Supplenmentary Figure 3. Results for multiple imputations (10 times) of missing data


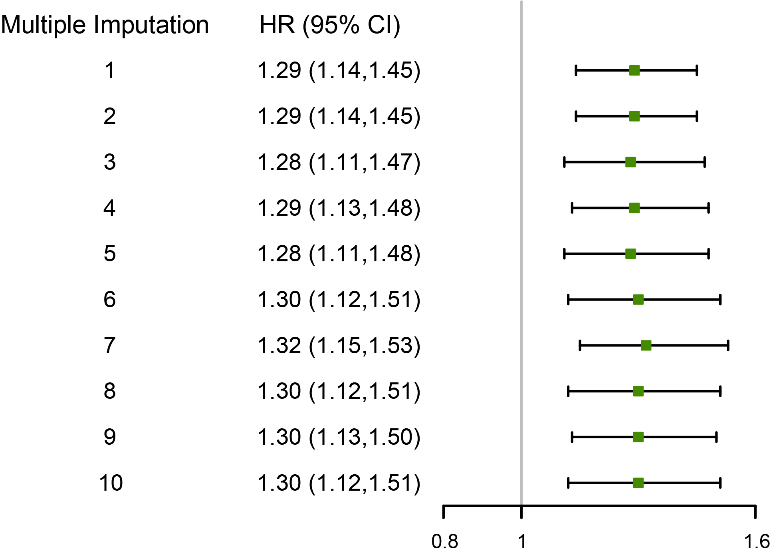


HRs were calculated to estimate the association between lnSII and all-cause mortality
